# Attitudes of health care professionals towards interprofessional teamwork in Ashanti Region, Ghana

**DOI:** 10.1186/s12909-023-04307-z

**Published:** 2023-05-08

**Authors:** Edward T. Dassah, Veronica M. Dzomeku, Betty R. Norman, Daniel Gyaase, Mercy N. A. Opare-Addo, Kwame O. Buabeng, Yaw Adu-Sarkodie

**Affiliations:** 1 Department of Population, Family and Reproductive Health, School of Public Health, Kwame Nkrumah University of Science and Technology, Kumasi, Ghana; 2 Department of Nursing, College of Health Sciences, Kwame Nkrumah University of Science and Technology, Kumasi, Ghana; 3 School of Medicine and Dentistry, Kwame Nkrumah University of Science and Technology, Kumasi, Ghana; 4 Injury Division, The George Institute for Global Health, University of New South Wales, Sydney, Australia; 5 Faculty of Pharmacy and Pharmaceutical Sciences, Kwame Nkrumah University of Science and Technology, Kumasi, Ghana

**Keywords:** Attitude, Exploratory factor analysis, Health professionals, HIV care, Ghana, Interprofessional training, Teamwork

## Abstract

**Background:**

Interprofessional collaboration ensures that high-quality health care is provided leading to improved health outcomes and provider satisfaction. Assessing the attitudes of health care professionals towards teamwork in Ghana is novel.

**Objective:**

To examine the attitudes of health care professionals towards interprofessional teamwork and assess specific attributes influencing these attitudes in the Ashanti region, prior to implementing an in-service interprofessional HIV training programme.

**Methods:**

A cross-sectional pre-training online survey using a modified Attitudes Toward Health Care Teams Scale was conducted among health care practitioners undergoing a two-day interactive interprofessional HIV training in Kumasi and Agogo from November 2019 to January 2020. Trainees were diverse health professional cadres selected from five hospitals in the Ashanti region of Ghana. Data was summarised using the mean and standard deviation for continuous variables, and frequencies and percentages for categorical variables. An exploratory factor analysis was conducted to categorise the 14 items of the modified attitudes scale. The Wilcoxon rank-sum (Mann–Whitney) and Kruskal–Wallis tests were used to test the mean attitude difference among the demographic characteristics. Statistical significance was set at *p* < 0.05.

**Results:**

Altogether, 302 health professionals completed the survey. The ages ranged from 20–58 years, mean age 27.96 years (standard deviation 5.90 years). Up to 95% of the trainees agreed with the 14 statements on the modified attitudes scale. Three factors were identified; “quality of care”, “team efficiency”, and “time constraint” with Cronbach’s alpha measures of 0.73, 0.50, and 0.45 respectively. The overall mean attitude score was 58.15 ± 6.28 (95% CI, 57.42–58.88). Attitude of health care professionals towards interdisciplinary teams for patient care varied significantly by age (*p* = 0.014), health profession cadre (*p* = 0.005), facility (*p* = 0.037), and professional experience (*p* = 0.034).

**Conclusion:**

Strengthening in-service interprofessional training for health practitioners especially early career professionals in the Ashanti region would be valuable.

**Supplementary Information:**

The online version contains supplementary material available at 10.1186/s12909-023-04307-z.

## Introduction

Interprofessional collaboration ensures that safe and effective health care is provided at all levels of the health care delivery system, leading to improved health outcomes, positive patient experiences, provider satisfaction, and efficient use of organisational resources [1, 2]. This is particularly important in resource-constrained settings with an increasing burden of long-term conditions with comorbidities such as HIV [1]. Negative attitudes toward other health professionals lead to poor communication among health care workers and provider dissatisfaction and negatively impacts on team-based care [3]. Interprofessional training can break down professional silos and promote collaborative practice, which is essential in delivering high quality team-based HIV care as well as the management of common major diseases, especially in sub-Saharan Africa [1]. Previous interprofessional training programmes among health care providers have reported improved participants’ attitudes, knowledge about their roles and responsibilities, as well as collaborative skills and behaviour towards teamwork [3, 4]. To strengthen interprofessional HIV training among health professionals in sub-Saharan Africa, the African Forum for Research and Education in Health (AFREhealth) in collaboration with the University of California, San Francisco developed and assisted the implementation of an interprofessional HIV training programme across 14 sub-Saharan African countries including Ghana [1].

Most professional health training programmes in Ghana are based on independent professional teaching, which could adversely affect interprofessional collaboration and cooperation among different health professions during health care delivery [2]. For instance, most nurses and midwives are trained in nursing and midwifery training colleges, which run nursing- and midwifery-specific and general courses such as entrepreneurship, with limited opportunities for interprofessional education [5, 6]. Moreover, in most Ghanaian universities where professional health programmes are offered in one college, interprofessional education among the different professions is lacking [7] or at best minimal [8]. For example, the College of Health Sciences of the Kwame Nkrumah University of Science and Technology has various schools and faculties which train health care professionals including medical doctors, dentists, pharmacists, nurses and midwives, laboratory scientists and physician assistants. These schools and faculties train their health cadres independently and interprofessional education within the college is largely lacking. Post-graduation, health care professionals may have in-service training together, but usually not in small teams with the appropriate mix of health professional cadres. A few studies have examined interprofessional education among health care professions in Ghana [7, 8]; there is a dearth of information on attitudes towards collaborative practice. Hence, assessing the attitudes of health care professionals towards teamwork in Ghana is novel. We aimed to examine the attitudes of health care professionals towards interprofessional teamwork and assess specific attributes influencing these attitudes in the Ashanti region, prior to implementing an in-service interprofessional HIV training programme.

## Methods

### Study design and population

A cross-sectional pre-training survey using a modified Attitudes Toward Health Care Teams Scale (ATHCTS)[9] was conducted among health care providers undergoing a two-day interactive interprofessional HIV training in Kumasi and Agogo from November 2019 to January 2020. The training comprised eight HIV specific modules reflecting commonly encountered clinical or programmatic challenges developed by a panel of leading health educators from across AFREhealth network and the University of California, San Francisco [1]. The main training was preceded by training of trainers to facilitate the subsequent training. Trainees were from the following health professions; Medicine and Dentistry, Pharmacy, Physician Assistantship, Nursing and Midwifery, and Medical Laboratory Sciences. Health care workers were selected from one teaching and five district hospitals in the Ashanti Region: Komfo Anokye Teaching Hospital (KATH), Maternal and Child Health Hospital and Suntreso Government Hospital, in Kumasi; Bekwai Municipal Hospital; Obuasi Government Hospital and Agogo Presbyterian Hospital. The health care professionals comprised new and old providers. New providers were newly qualified health care professionals within 12 months of graduation. Hence the new providers included house officers, interns, and rotation nurses/midwives. Old providers were professionals who had been working for more than 12 months post qualification. The training was conducted in English; all trainees were proficient in English.

### Study sites

KATH is the second-largest hospital in Ghana, and one of the main tertiary referral health facilities in the northern sector of the country. It is a 1,200-bed capacity hospital with 12 clinical directorates including the Directorate of Medicine. The hospital trains various categories of health professionals including nurses and midwives, medical and dental students, pharmacy students, laboratory scientists, house officers, and postgraduate resident doctors. The Department of Medicine has several sub-specialties including the Infectious Disease Unit which has a specialized HIV clinic. Patients at the HIV clinic are managed by a team of physicians, nurses, pharmacists, and other health care providers. HIV clinics were held twice and three times a week for adults and children respectively. An average of 300 and 80 new adult and childhood cases of HIV were attended to at the clinic each year. Other directorates such as Obstetrics and Gynaecology also attend to cases in conjunction with physicians from the unit. The other hospitals were the first referral hospitals (district hospitals) in their respective districts/metropolitan area. All the hospitals had specific clinics for people living with HIV with each facility/clinic attending to an average of 1000–2500 cases (about 100–300 new cases) a year. Except for the Agogo Presbyterian Hospital, all the other hospitals were being supported by the (United States) President’s Emergency Fund for AIDS Relief (PEPFAR) at the time.

### Study procedures

We engaged key stakeholders in the partner institutions and facilities from the onset and throughout the planning and implementation of the programme. All health professionals, house officers and interns of the five health professions in KATH, and health care professionals involved in HIV care in the selected hospitals were eligible for the training. Within KATH, the number of participants from each profession was selected by the programme/unit head in proportion to their number in the unit. For health care workers in the selected hospitals, 12 professionals working in HIV care were selected by their unit heads for the training.

To assess trainees’ attitudes towards interprofessional health care teams, participants were asked to complete an online Google form (Google, Inc., Mountain View, CA, USA) pre-training survey in English using a modified ATHCTS with 14 items by Curran et al. [9]. This modified scale was adapted from Heinemann et al. [10], who identified three main factors as influencing attitudes namely quality of care, cost of team care and physician centrality, comprising 14, seven and six items respectively [10]. For the modified ATHCTS, Curran et al. selected 11 items from the quality of care factor and three items from the costs of team care factor as appropriate for pre-licensure students with little or no experience with items relating to physician centrality [9]. We chose the 14-item modified scale since that is recommended for assessing the attitudes towards health care teams among a wide variety of health professions [9, 11, 12]. The survey was typically completed within the 24 h preceding the training. The survey tool included sections on trainee characteristics (age, gender, profession, health facility, and professional experience) and the 14 items on the adapted ATHCTS. Responses to the 14 items were scored on a five-point Likert scale ranging from one (strongly disagree) to five (strongly agree). Three items regarding time constraints which are worded such that agreement represents negative attitudes were reverse scored. Total scores ranged from 14 to 70 with higher scores indicating more positive attitudes toward interprofessional health care teams.

### Statistical analysis

Data was summarised using descriptive statistics of mean and standard deviation for continuous variables, and frequencies and percentages for categorical variables. An exploratory factor analysis was conducted to categorise the 14 items of the modified ATHCTS. The suitability of the data for factor analysis was assessed. The correlation matrix showed several coefficients were ≥ 0.3 indicating high correlation among items for factor analysis [13]. The Bartlett test of sphericity (*p* < 0.001) and Kaiser–Meyer–Olkin measure of sampling adequacy (0.79) confirmed strong correlation for application of dimensionality reduction [13, 14] among the 14 items in the modified ATHCTS, and the adequacy of the sample for factor analysis respectively. Factor extraction was performed using principal component analysis and factors with eigenvalues > 1 (Kaiser’s criterion) were retained. Factor rotation was performed using Varimax rotation and items with factor loadings of at least 0.4 were considered to contribute to that factor [14, 15]. Internal consistency was assessed using Cronbach’s alpha with a threshold of 0.7 [16]. The overall Cronbach’s alpha for the 14 modified items was 0.71. The overall mean score was estimated by adding all 14 items; all negatively worded statements were reverse scored. Shapiro–Wilk normality test was used to check the normality of the data; the overall attitude score was not normally distributed (*p* = 0.005). Hence, the Wilcoxon rank-sum (Mann–Whitney) and Kruskal–Wallis tests were used to test the mean attitude difference among the demographic characteristics. All statistical analyses were performed using Stata 17.0 (StataCorp, Texas, USA), and *p* < 0.05 was considered statistically significant.

## Results

Three hundred and sixty-two health care professionals were trained within the period and 302 (83.43%) completed the pre-training online survey. Hence, 302 health professionals were recruited into the study. Table 1 presents the demographic characteristics of the respondents. The mean age was 27.96 years (standard deviation 5.90 years), range of 20–58 years and about 70% were in their twenties. Most of the practitioners were males (59.14%), and nearly three-quarters (72.5%) were health workers at KATH. About 70% of the health workers were either medical doctors or nurses/midwives, with the other professions contributing less than 15% each. Majority (71.52%) of the trainees were new providers (interns/house officers and rotation nurses/midwives). Further analysis of the professional experience, age group, health facility, and profession revealed the following: Less than 10% of new providers and over 80% of old providers were at least 30 years old; all but five new providers were at KATH and all selected providers from PEPFAR-assisted district hospitals were old providers; 70% of physician assistants were old providers, while the majority of all the other professions were new providers (nurses/midwives and pharmacists had a higher proportion of old providers) (see supplementary table).

**Table 1:** Demographic characteristics of trainees

Characteristics	Frequency *N* = 302	Percentage (%)
Age group (years)		
20–24	105	34.77
25–29	109	36.09
30–34	46	15.23
35 +	42	13.91
Mean ± SD	27.96 ± 5.90	
Sex		
Male	178	58.94
Female	124	41.06
Facility		
Agogo Presby Hospital	27	8.94
Komfo Anokye Teaching Hospital	219	72.52
PEPFAR- assisted district hospitals	56	18.54
Health profession cadre		
Medical	96	31.79
Laboratory	43	14.24
Nursing/Midwifery	118	39.07
Pharmacy	35	11.59
Physician Assistantship	10	3.31
Professional experience^a^		
New provider	216	71.52
Old provider	86	28.48

PEPFAR-President’s Emergency Fund for AIDS Relief; PEPFAR-assisted district hospitals: Bekwai Municipal, Maternal and Child Health, Obuasi Government and Suntreso Government Hospitals; *SD* standard deviation;

^a^New provider, interns/house officers and rotation nurses/midwives; old provider, health providers with professional experience more than one year

Table 2 summarises the trainees' responses to the 14 items on the adapted ATHCTS. Analysis of participants’ responses to each statement on the scale revealed that 11–95% agreed (or strongly agreed) with the statements. Generally, higher proportions of the participants agreed or strongly agreed with the positively worded statements while relatively fewer positive responses were associated with the negatively worded statements. Overall, up to 95% (range 59.34%-95.02%) of participants agreed (or strongly agreed) with the positively worded statements with less than a third (range 2.66%-31.67%) being neutral in their responses to these statements. For the negatively worded statements, up to 74.5% (range 29.29%-74.50%) disagreed (or strongly disagreed) with these statements, with two of the statements having the highest neutral responses of 37% and 43.10%.

**Table 2:** Health worker's responses to the 14 items of the Attitudes Toward Health Care Team Scale

	Statements	SD	D	N	A	SA
1	The team approach permits health professionals to meet the needs of family caregivers as well as patients	0.67	1.34	20.81	26.17	51.01
2	Working on a team keeps most health professionals enthusiastic and interested in their jobs	0.66	0.66	16.56	24.17	57.95
3	Having to report observations to the team helps team members better understand the work of other health professionals	0.67	1.01	15.44	26.51	56.38
4	The team approach makes the delivery of care more efficient	1.01	1.35	5.39	17.85	74.41
5	The team approach improves the quality of care to patients	1.66	0.66	2.66	15.95	79.07
6	Patients receiving team care are more likely than other patients to be treated as whole persons	4.35	3.01	21.07	25.42	46.15
7	The give and take among team members helps them make better patient care decisions	6.00	3.67	24.00	19.00	47.33
8	Hospital patients who receive team care are better prepared for discharge than other patients	2.33	3.33	16.33	24.67	53.33
9	In most instances, the time required for team meetings could be better spent in other ways^a^	27.67	18.67	37.00	8.33	8.33
10	Developing a patient care plan with other team members avoids errors in delivering care	1.33	1.33	12.29	25.25	59.80
11	Team meetings foster communication among team members from different disciplines	1.66	0.99	4.64	17.88	74.83
12	Health professionals working on teams are more responsive than others to the emotional and financial needs of patients	6.67	2.33	31.67	28.67	30.67
13	Working in teams unnecessarily complicates things most of the time^a^	55.63	18.87	14.24	4.64	6.62
14	Developing an interdisciplinary patient care plan is excessively time-consuming^a^	14.81	14.48	43.10	16.84	10.77

*A* Agree, *D *Disagree, *N *Neutral, *SA *Strongly agree, *SD *Strongly disagree

^a^Negatively worded statements were reverse scored to calculate

The results of the exploratory factor analysis are presented in Table 3. Three factors were identified as; “quality of care” (factor 1), “team efficiency” (factor 2), and “time constraint” (factor 3) with Cronbach’s alpha measures of 0.73, 0.50, and 0.45 respectively. The percentage of explained variance in the three-factor structure was 53.7%. For factor 1, the dominant statements were; having to report observations to the team helps team members better understand the work of other health professionals (with a factor loading of 0.80) and two other statements with factor loadings of 0.76 and 0.67. The strongest statements in factors 2 and 3 had factor loadings of 0.64 and 0.74 respectively.

**Table 3:** Varimax rotation with three factors for responding sample

Item	Statement	Factor Loading		
		**I**	**II**	**III**
		**α = 0.73**	**α = 0.50**	**α = 0.45**
3	Having to report observations to the team helps team members better understand the work of other health professionals	0.80	-0.05	0.06
1	The team approach permits health professionals to meet the needs of family caregivers as well as patients	0.76	0.15	0.02
4	The team approach makes the delivery of care more efficient	0.67	0.11	0.06
7	The give and take among team members help them make better patient care decisions	0.54	0.18	0.02
2	Working on a team keeps most health professionals enthusiastic and interested in their jobs	0.53	0.38	0.04
11	Team meetings foster communication among team members from different disciplines	0.04	0.09	0.05
5	The team approach improves the quality of care to patients	0.19	0.06	-0.02
10	Developing a patient care plan with other team members avoids errors in delivering care	0.34	0.23	-0.01
6	Patients receiving team care are more likely than other patients to be treated as whole persons	-0.08	0.64	0.04
12	Health professionals working on teams are more responsive than others to the emotional and financial needs of patients	0.35	0.64	-0.07
8	Hospital patients who receive team care are better prepared for discharge than other patients	0.30	0.56	0.14
14	Developing an interdisciplinary patient care plan is excessively time consuming^a^	-0.02	-0.10	0.74
9	In most instances, the time required for team meetings could be better spent in other ways^a^	0.09	0.19	0.74
13	Working in teams unnecessarily complicates things most of the time^a^	0.11	-0.44	0.50

^a^Negatively worded statements were reverse scored to calculate; α, Cronbach’s alpha

Table 4 compares the mean ATHCTS scores for various categories of the demographic characteristics of the participants. The overall mean score was 58.15 ± 6.28 (95% CI, 57.42–58.88). The attitude of health care professionals towards interdisciplinary teams for patient care differed significantly by age (*p* = 0.014), health profession cadre (*p* = 0.005), health facility (*p* = 0.037), and professional experience (*p* = 0.034). Older health workers who were at least 35 years old were more likely to have a more positive attitude towards teamwork, as were physician assistants, health workers from the PEPFAR-assisted district hospitals, and old trainees who had been working for more than one year.

**Table 4:** Comparison of overall ATHCTS mean scores by demographic characteristics

Variable	Attitude score (Mean ± SD)	Statistic^a^	*P*-value
Age group (years)		10.567	0.014
20 – 24	57.82 ± 5.79		
25 – 29	57.56 ± 6.62		
30 – 34	57.46 ± 6.37		
35 +	61.28 ± 5.68		
Gender		478,360.39	0.405*
Female	58.35 ± 6.40		
Male	57.92 ± 6.12		
Facility		6.575	0.037
Agogo Presby Hospital	57.85 ± 6.46		
Komfo Anokye Teaching Hospital	57.68 ± 6.08		
PEPFAR-assisted district hospitals	60.19 ± 6.66		
Health profession cadre		14.798	0.005
Medical	56.83 ± 6.31		
Laboratory	56.81 ± 7.02		
Nursing/Midwifery	59.10 ± 5.26		
Pharmacy	59 ± 7.10		
Physician Assistantship	63 ± 6.93		
Professional experience		405,591.72	0.034*
New trainees	57.63 ± 6.10		
Old trainees	59.46 ± 6.55		
All trainees	58.15 ± 6.28	(57.42, 58.88)	

New provider, interns/house officers and rotation nurses/midwives; old provider, health providers with professional experience more than one year

*ATHCTS* Attitudes Toward Health Care Teams Scale, PEPFAR-assisted district hospitals: Bekwai Municipal, Maternal and Child Health, Obuasi Government and Suntreso Government Hospitals

^a^Data presented as statistic or (95% confidence interval)

^*^Mann–Whitney test, and Kruskal–Wallis’ test for the others

## Discussion

This study assessed the attitudes of health care professionals towards teamwork in one region in Ghana, prior to an interprofessional HIV training programme. Generally, most participants agreed (or strongly agreed) with the positively worded statements with relatively fewer agreeing with the three negatively worded statements on the 14-item scale. Three factors were identified in the factor analysis: quality care, team efficiency, and time constraint. The attitude of health care professionals towards interprofessional teamwork differed significantly by age, profession, facility, and professional experience.

Analysis of the trainees’ responses to the 14 items on the scale revealed that the majority of the practitioners felt that interprofessional practice would be beneficial to the patient. Most professionals disagreed with the negatively worded statements on the time requirements for interprofessional collaboration, except for the statement on developing an interdisciplinary patient care plan where over 40% were neutral in their responses, indicating they were not certain of the time demands of this care plan prior to the training. The former suggests that the trainees did not find interprofessional training to be complicated and the time spent on interprofessional training and team-based care was worthwhile. It is conceivable that the uncertainty regarding the time requirement for a multidisciplinary patient care plan could change to positive attitudes when the practitioners are actually involved in developing this patient care plan [17]. Overall, these findings indicate that the participants had a positive attitude toward interprofessional teamwork, suggesting that these professionals are likely to embrace interprofessional training and collaborative practice. These positive attitudes are also supported by the mean attitude scores of the trainees (Table 4), which increased with years of professional experience. As observed in one study [3], health care practitioners are usually exposed to interprofessional practice leading to a better appreciation of the role of team collaboration in achieving work efficiency and improved health outcomes for patients. This will result in positive attitudes towards interprofessional teamwork. Interprofessional collaboration by health care workers also has implications for the training of health professional students and residents; it affords trainees the opportunity to acquire appropriate interprofessional skills from experienced practitioners [18]. These competencies are likely to enhance interprofessional collaboration when they graduate as professionals in the future [9]. Since training improves health workers' attitudes towards teamwork, team skills, and behaviour [3, 4], these observed positive attitudes are likely to be augmented after our interprofessional HIV training. Given the positive attitude of these professionals towards interdisciplinary teamwork, it is probably about time to consider implementing interprofessional education in the pre-service training curriculum of the various health professions and complement this with in-service training for practising professionals.

Exploratory factor analysis of the modified ATHCTS [9] yielded three subscales; quality of care, team efficiency, and time constraints. These subscales were reported in previous studies [9, 11, 12] which used the same modified ATHCTS. The quality of care subscale was consistent and also had the highest reported Cronbach’s alpha (0.72–0.82), across the three previous studies [9, 11, 12] and our study, indicating that participants’ response values for the set of statements relating to this subscale were acceptably consistent across the different study settings. Again, most participants across the different settings perceived that patients could receive quality care through effective collaboration among health care professionals [19]. Two of the previous studies identified team efficiency [11, 12] and one reported on time constraints [9] as factors. Together, these findings emphasize the contribution of these subscales to health care professionals’ attitudes towards collaborative practice across different settings.

Consistent with the results of previous studies [3, 20], we observed that attitudes towards teamwork improved with professional experience; old providers (health professionals who had been working for more than a year) had better attitudes compared to new providers, as were older professionals (> 34 years) compared to younger providers. Older professionals were more likely to have worked longer. The differences observed across facilities and professions could be partly explained by the number of years of professional experience. All the providers from the PEPFAR-supported district hospitals and majority of providers from Agogo Presby Hospital were old providers while almost all the new providers were from KATH. Thus, accounting for the lowest reported mean ATHCTS score from trainees at KATH. Among the different health professionals, physician assistants had the highest mean ATHCTS scores followed by nurses/midwives and then pharmacists mainly due to the proportion of old providers among these cadres; 70% of physician assistants were old providers. It is quite conceivable that most team skills of new providers may have been acquired during pre-service training. Together, these findings suggest that most providers acquired team skills on the job (which improved with years of work experience) rather than during pre-service training, emphasizing the need for implementing/strengthening team skills training during the pre-service period. However, this may differ across professions. In one study in Germany, physicians acquired more interprofessional skills including teamwork through work experience rather than pre-service training, while the converse was true for nurses [21]. Interprofessional training has been shown to improve interprofessional competencies including team skills of practicing health professionals [22, 23].

Assessing the attitude of health care professionals towards collaborative practice in HIV care is novel in Ghana, and our participants were selected across the common health care professions in the country. The study had some limitations. Attitudes towards interprofessional teamwork were self-reported and are subject to social desirability bias. The number of practitioners from the various professions and facilities was based on predetermined numbers of old and new providers from the various facilities (which was skewed towards newly qualified professionals mainly from KATH), and does not reflect the distributions of these professionals involved in HIV care across facilities in the country. In addition, health care providers working in HIV care in the various hospitals were chosen by their unit heads (at their discretion) and selection bias cannot be excluded. Therefore, the results of this study which were largely from a single tertiary facility and a few district-level hospitals cannot be generalized to health facilities especially lower-level facilities in the country. Although prior exposure to interprofessional education/training could influence the attitudes of the practitioners, over 70% of the trainees were newly qualified professionals without previous formal experience. Finally, as a commonly reported limitation of teamwork attitude studies [24], these observed positive attitudes may not necessarily translate into collaborative practice.

## Conclusion

Health care professionals in the Ashanti region of Ghana had a positive attitude towards collaborative practice, which differed significantly by age, facility, health profession cadre, and professional experience. Interventions are required to strengthen in-service interprofessional training for health practitioners, especially early career professionals. Investigating whether these positive team attitudes translate into collaborative health care delivery for patients would be worthwhile.

## Supplementary Information

**Additional file 1:** **Supplementary Table.** Demographic, facility and professional categories and professional experience.

## Data Availability

The datasets used and analysed during the current study are available from the corresponding author on reasonable request.

## Abbreviations

AFREhealth: African Forum for Research and Education in Health
ATHCTS: Attitudes Toward Health Care Teams Scale
HIV: Human immunodeficiency virus
KATH: Komfo Anokye Teaching Hospital
PEPFAR: President’s Emergency Fund for AIDS Relief
