# Supplementary material for: Attitudes of health care professionals towards interprofessional teamwork in Ashanti Region, Ghana
Source: BMC Med Educ. 2023 May 8;23:319. doi: 10.1186/s12909-023-04307-z (PMC10165774; doi:10.1186/s12909-023-04307-z)
Supplement: Supplementary file 1 — Additional file 1: Supplementary Table. Demographic, facility and professional categories and professional experience. [file 12909_2023_4307_MOESM1_ESM.docx]

**Supplementary Table:** **Demographic, facility and professional categories and professional experience**

| **Variable** | **Professional experience** | | |
| --- | --- | --- | --- |
|  | **New provider**  **(n=216)** | **Old provider**  **(n=86)** | **Total**  **(N=302)** |
|  | **n (%)** | **n (%)** | **n (%)** |
| **Age group (years)** |  |  |  |
| 20-24 | 104 (48.15) | 1 (1.16) | 105 (34.77) |
| 25-29 | 93 (43.06) | 16 (18.60) | 109 (36.09) |
| 30-34 | 13 (6.02) | 33 (38.37) | 46 (15.23) |
| 35+ | 6 (2.78) | 36 (41.86) | 42 (13.91) |
| **Facility** |  |  |  |
| Agogo Presby Hospital | 5 (2.31) | 22 (25.58) | 27 (8.94) |
| Komfo Anokye Teaching Hospital | 211 (97.69) | 20 (23.26) | 231 (76.49) |
| PEPFAR-assisted district hospitals | 0 (0.00) | 44 (51.16) | 44 (14.57) |
| **Health profession cadre** |  |  |  |
| Medical | 89 (41.20) | 7 (8.14) | 96 (31.79) |
| Laboratory | 36 (16.67) | 7 (8.14) | 43 (14.24) |
| Nursing/Midwifery | 65 (30.09) | 53 (61.63) | 118 (39.07) |
| Pharmacy | 23 (10.65) | 12 (13.95) | 35 (11.59) |
| Physician Assistantship | 3 (1.39) | 7 (8.14) | 10 (3.31) |

PEPFAR-assisted district hospitals: Bekwai Municipal, Maternal and Child Health, Obuasi Government and Suntreso Government Hospitals
